# Sea Blue Histiocytosis Concordant With Immune Thrombocytopenic Purpura

**DOI:** 10.7759/cureus.10396

**Published:** 2020-09-11

**Authors:** Arshia Bhardwaj, Monica Gupta, Anita Tahlan, Sanjay D'Cruz, Saurabh Gaba

**Affiliations:** 1 General Medicine, Government Medical College and Hospital, Chandigarh, IND; 2 Pathology, Government Medical College and Hospital, Chandigarh, IND

**Keywords:** sea blue histiocytosis, immune thrombocytopenic purpura

## Abstract

Sea blue histiocytosis is an unusual bone marrow finding in many haematological conditions or lipid metabolic diseases that by itself may not carry any prognostic value. It may occur rarely as a primary genetic clinical syndrome characterized by splenomegaly, hypertriglyceridemia and thrombocytopenia. More commonly, the presence of these lipid-laden blue-stained macrophages indicates an underlying condition characterized by increased bone marrow precursor cell turnover due to myeloproliferative conditions or ineffective erythropoiesis. Rarely may they be observed in cases of immune thrombocytopenic purpura (ITP) incidentally due to rapid megakaryocytic turnover. Sea blue histiocytosis should prompt the clinician to evaluate the patient for more sinister conditions such as myelodysplastic syndrome or infiltrative disorders.

## Introduction

Bone marrow is usually carried out in immune thrombocytopenic purpura (ITP) to rule out hypomegakaryocytic thrombocytopenic diseases such as aplastic anemia. The bone marrow aspirate in ITP is characterized by increased megakaryocytes though occasionally they may be normal or rarely decreased. The erythroid and myeloid lineages are essentially normal in ITP. Rarely the bone marrow may reveal large lipid-laden macrophages called sea blue histiocytes that are common cytological feature of chronic myelogenous leukemia and myelodysplastic syndromes [1].

## Case presentation

A 47-year-old male presented with complaints of generalized weakness of one-month duration following an episode of short-lasting fever a month back. He had no anorexia, weight loss, bony or joint pains, skin rash, pedal edema, abdominal distension, chest pain or visual complaints. There were no gait abnormalities or jerky limb movements, hearing loss. He had no past history of easy bruising, recurrent infections or any major surgery requiring total parenteral nutrition. There was no relevant family history of any heritable or chronic illnesses. On examination, he was well-built and no petechiae, ecchymosis, mucocutaneous bleed or any rash or subcutaneous nodules were discernible. Sternal tenderness, lymphadenopathy and organomegaly were absent. The cardiovascular, neurological and respiratory examination was within normal limits.

His haemogram revealed haemoglobin 15.4 g/dL, haematocrit 45%, RBC count 5.31 x 10^12^/L with normal indices and reticulocyte count 2.85%, total leucocyte count 8.1 x 10^9^/L with normal differential, and platelet count of 35 x 10^9^/L. Peripheral blood smear was normocytic normochromic with reduced platelets and a few giant ones. The platelet count on day two and three fell down to 14x10^9^/L and 12x10^9^/L, respectively. His fasting blood sugar was incidentally detected to 174 mg/dL and HbA1C was 8.5 %. Serum electrolytes, complete lipid profile including triglycerides, coagulation profile, urine analysis, renal and liver function tests were within the normal range. In view of thrombocytopenia, serological tests for dengue, malaria, scrub typhus, leptospirosis, chikungunya and brucella were negative. Hepatitis B, hepatitis C and HIV serologies were negative. Autoimmune profile (anti-nuclear antibodies, rheumatoid factor, anti-thyroid peroxidase antibody, anti-tissue transglutaminase antibody) was negative. On ultrasonography of abdomen, liver measured 13.2 cm with normal echotexture and spleen measured 8.7 cm along its long axis (not enlarged).

**Figure 1:**
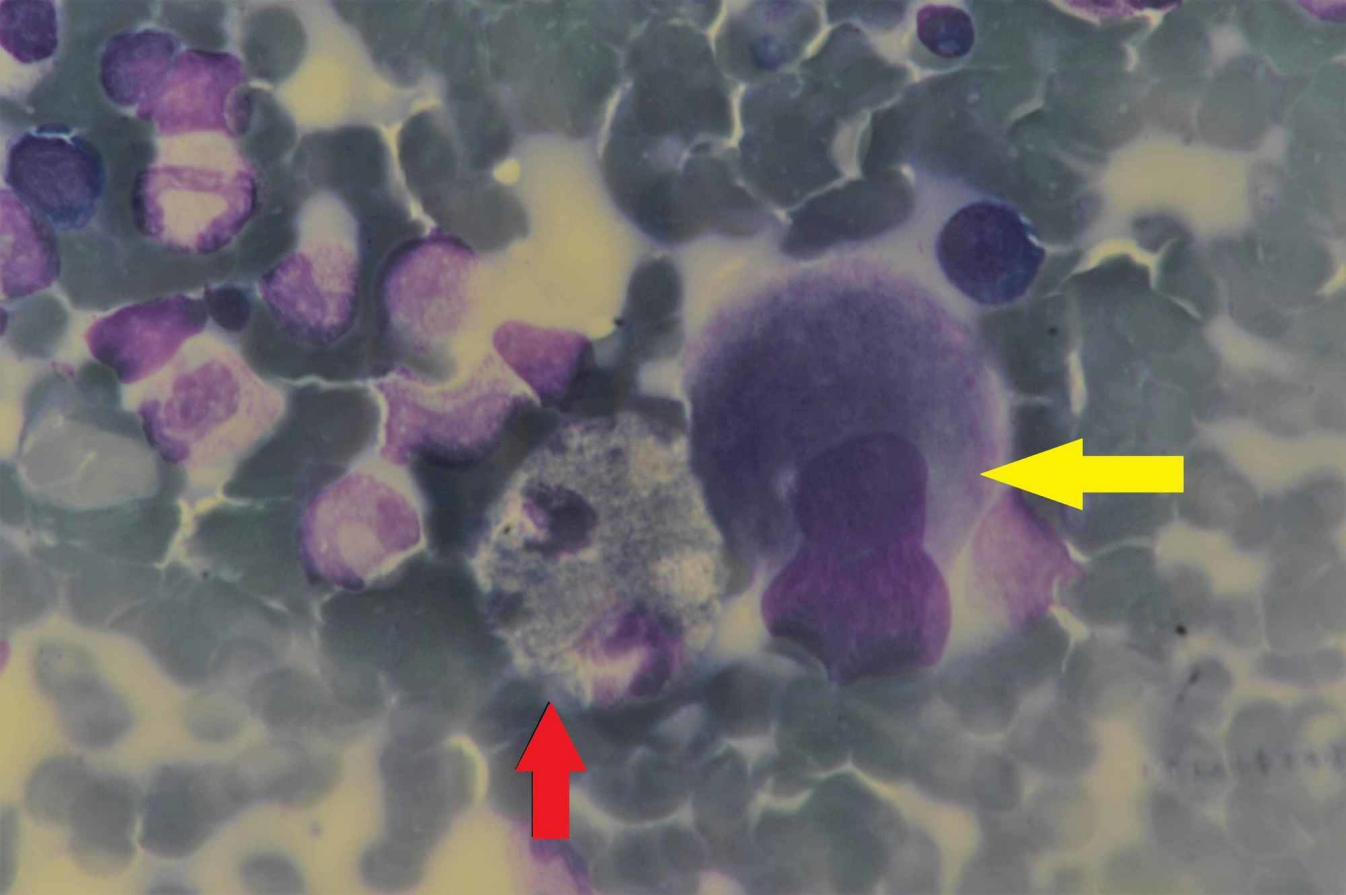
Bone marrow aspirate (May-Grünwald-Giemsa; 100x) showing hypolobated megakaryocytes (yellow arrow) and sea blue histiocyte (red arrow)

Considering the clinically possibility of immune thrombocytopenic purpura tentatively in view of a normal spleen size and isolated thrombocytopenia, a bone marrow aspirate and biopsy was carried out. Here it is important to highlight that the differential diagnosis of thrombocytopenia are exhaustive in a tropical setting with acute febrile illnesses and bone marrow infiltrative disorders leading the list. The marrow in our patient was normocellular for age with adequate representation of myeloid and erythroid lineages. The megakaryocytic series was hyperplastic with many hypolobated megakaryocytes (Figure 1).

**Figure 2:**
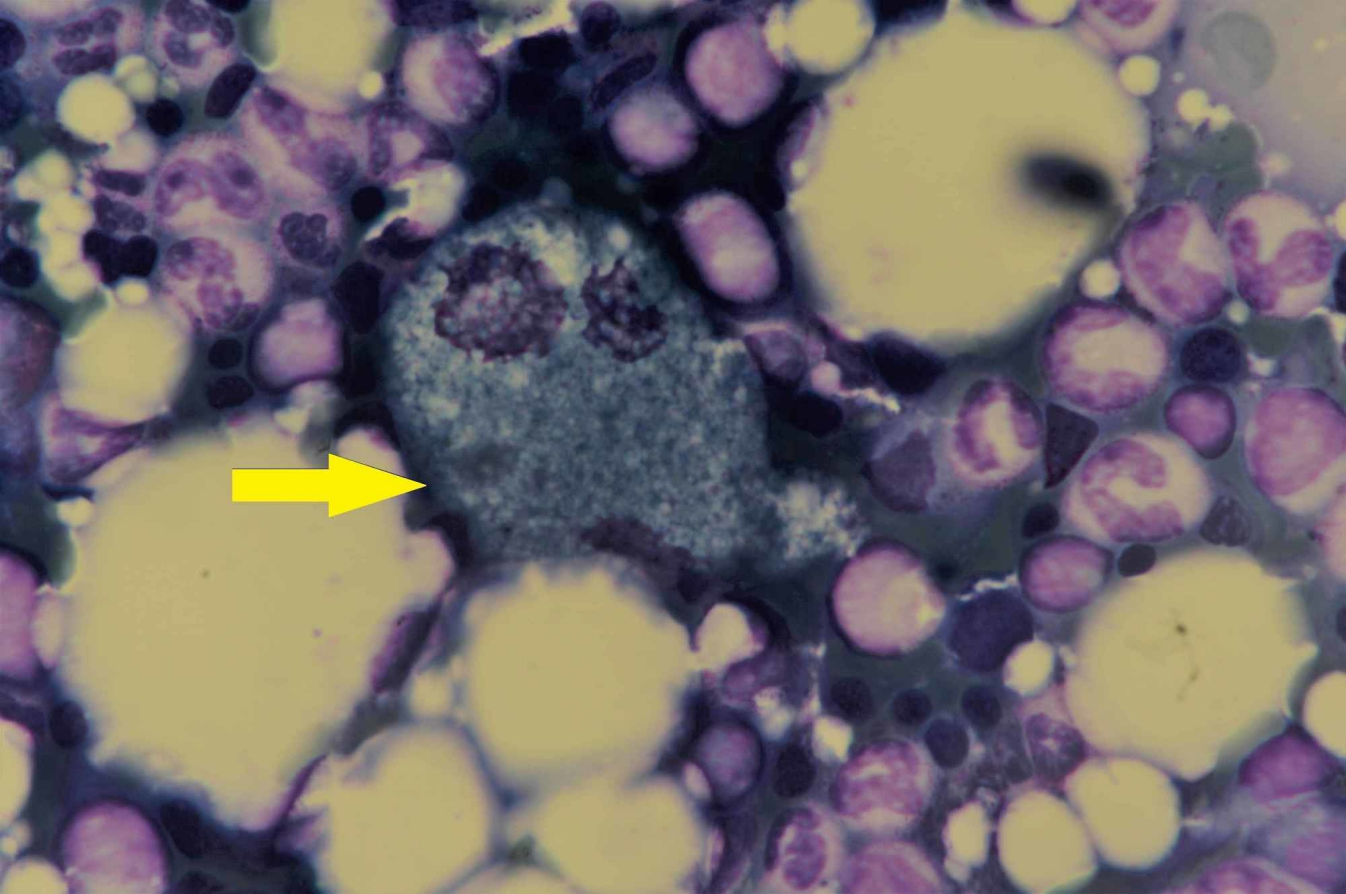
Bone marrow aspirate (May-Grünwald-Giemsa; 100x) showing typical sea blue histiocyte (yellow arrow) surrounded by marrow elements

In addition, there was a fascinating observation of scattered and small focal collections of histiocytes, some of which had a deep blue granular cytoplasm with central or eccentrically placed nuclei, conforming to the morphology of sea blue histiocytes (Figure 2).

**Figure 3:**
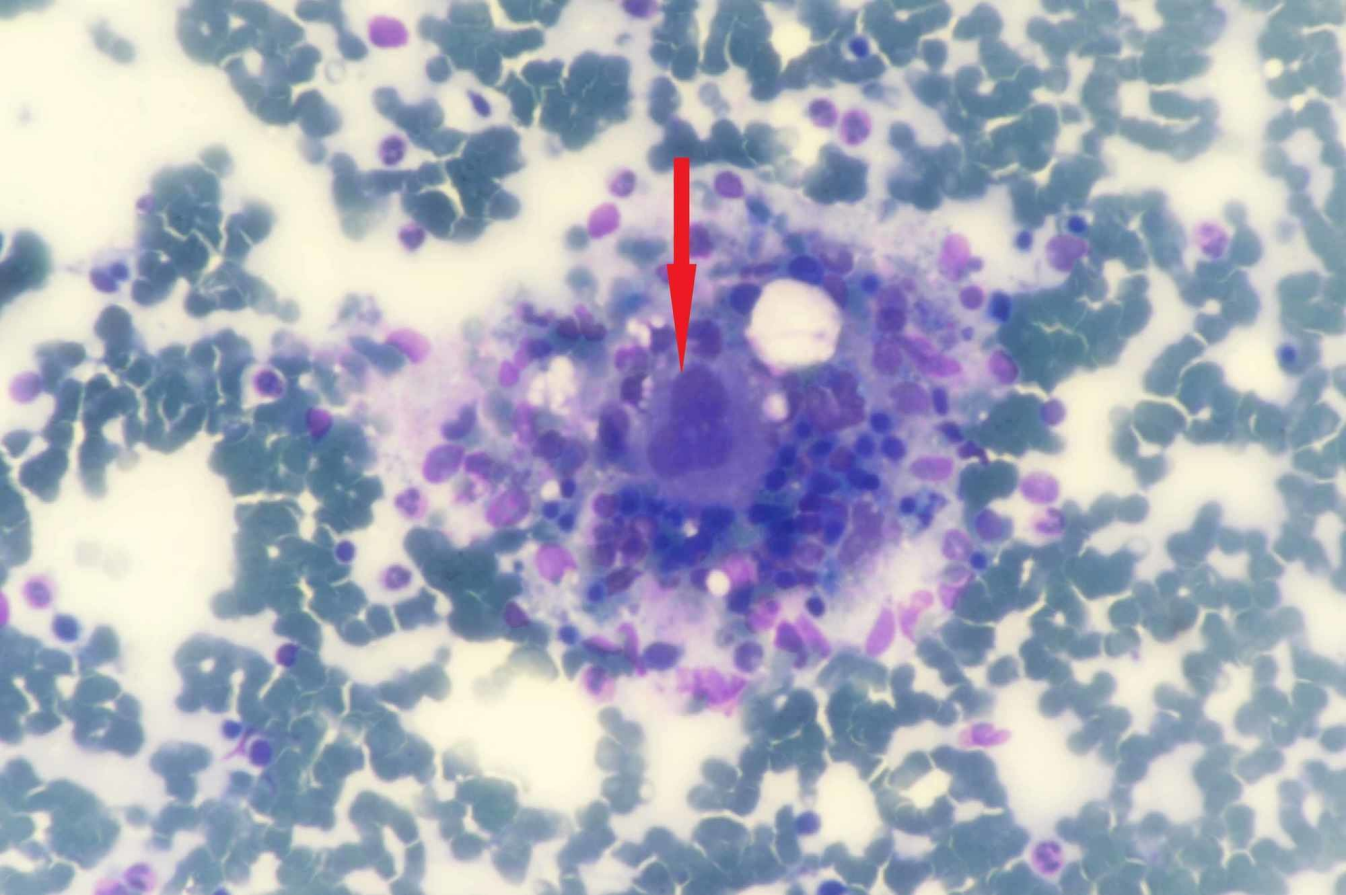
Trephine biopsy (40x) depicting megakaryocyte (red arrow) surrounded by histiocytes and haemopoetic cells

Sudan-Black B and Perl’s stains were negative. Trephine biopsy also depicted megakaryocytic hyperplasia with scattered and focal collections of histiocytes (Figure 3).

Stains for acid-fast bacilli and fungus were negative. There was no evidence of any clonal granulocytic proliferation. Cytogenetic studies did not reveal any abnormalities.

The various causes of secondary thrombocytopenia were excluded and a working diagnosis of newly diagnosed ITP was made in view of clinical and haematological findings. Patient was given a five-day course of intravenous methylprednisolone (1 gram/day) with close monitoring of his blood glucose levels. His platelet counts gradually improved by day 7 and he was discharged on oral prednisolone 1 mg/kg/day and pre-mixed human insulin. Patient continued prednisolone at this dosage for three weeks and then it was tapered at the rate of 5 mg per week. His platelet counts were 225 x 10^9^/L at one month of cessation of steroids.

## Discussion

Sea blue histiocytes may be recognizable in a primary rare genetic syndrome or as a secondary phenomenon in many haematological, lipid and ceroid metabolic and miscellaneous conditions. They may be observed in normal bone marrow aspirates, as well as with prolonged parenteral nutrition with fat emulsions and therapy with liposomal amphotericin B. Presence of sea blue histiocytes (SBH) does not signify a distinct entity and is more simply a morphological curiosity for identifying the underlying disease. They are a common cytological feature of marrow hyper-cellularity states like myeloproliferative diseases (particularly chronic myelogenous leukemia), lymphomas and myelodysplastic syndromes [1].

Besides ITP as in our patient, they could be a secondary finding associated with a wide range of non-malignant disorders where bone marrow has rapid cell turnover and infiltration as seen in thalassemia, severe autoimmune neutropenia, lipid storage diseases such as Gaucher disease and Niemann-Pick disease, and in patients receiving prolonged total parenteral nutrition and isolated hypertriglyceridemia [2-5]. Even rarely, SBH can be seen in a genetic syndrome variably called as primary or idiopathic sea‑blue histiocytosis or inherited lipemic splenomegaly, characterized by purpura, hypertriglyceridemia, thrombocytopenia, splenomegaly and progressive liver failure [6].

Sea blue histiocytes are lipid-laden macrophages which appear deep blue on Wright and Giemsa staining in bone marrow. These can also be found in other organs like spleen, liver, lung, lymph nodes, tonsils and skin. It was Rywlin et al. who deciphered that the blue-stain material was ceroid, a product of oxidation and polymerization of unsaturated lipids [7]. In ITP, an increased megakaryocytic production in the marrow generates an increased histiocytic activity and subsequent deposition of phospholipids in the macrophages, as a result, sea blue histiocytes are formed.

## Conclusions

ITP is associated with rapid turnover of megakaryocytic population as seen in our case. Sea blue histiocytes is a peculiar finding described in the setting of high rates of intramedullary cell turnover. Presence of sea blue histiocytes in bone marrow kindles the need to search for the causative haematological or infiltrative disorder. Our patient did not have any alternative diagnosis than ITP as confirmed by bone marrow biopsy and radiological investigations.
